# Supplementary material for: Effect of Copper Sulphate and Cadmium Chloride on Non-Human Primate Sperm Function In Vitro
Source: Int J Environ Res Public Health. 2021 Jun 8;18(12):6200. doi: 10.3390/ijerph18126200 (PMC8228149; doi:10.3390/ijerph18126200)
Supplement: Supplementary file 1 [file ijerph-18-06200-s001.zip › ijerph-1197319-supplementary.pdf]

Supplementary Material: IJERPH - 1197319

Table S1: Vervet sperm motility parameters over 75 minutes

Time	Sample size	Total Motility	Progressive Motility	Rapid Spermatozoa	Medium Spermatozoa	Slow Spermatozoa
(min)	n	(%)	(%)	(%)	(%)	(%)
15	5	86.14 ± 7.51	58.54 ± 21.88	54.29 ± 23.85	11.86 ± 10.29	19.99 ± 14.45
30	5	72.23 ± 18.08	40.99 ± 20.18	35.94 ± 20.11	10.78 ± 7.94	25.50 ± 12.99
45	5	68.46 ± 15.91	35.54 ± 15.49	30.75 ± 14.56	10.90 ± 5.31	26.81 ± 12.86
60	4	67.87 ± 15.81	26.39 ± 16.62	21.16 ± 14.85	11.80 ± 5.53	34.90 ± 14.17
75	4	55.74 ± 23.02	24.38 ± 23.65	19.60 ± 23.68	10.39 ± 7.47	25.75 ± 17.67

Table S2: Vervet sperm kinematic parameters over 75 minutes

Time	Sample size	VCL	VSL	VAP	LIN	STR	WOB	ALH	BCF
(min)	n	(μm/s)	(μm/s)	(μm/s)	(%)	(%)	(%)	(μm)	(Hz)
15	5	206.88 ±98.91	140.06 ±94.35	172.63 ±102.86	62.85 ±13.66	78.49 ±6.53	79.44 ±10.96	2.94 ±0.89	12.34 ±2.02
30	5	153.70 ±60.02	95.63 ±56.56	147.87 ±62.28	59.69 ±12.14	76.10 ±7.49	77.88 ±8.73	2.86 ±0.76	14.08 ±2.62
45	5	132.45 ±39.81	78.80 ± 8.54	104.25 ±42.43	57.92 ±12.25	74.60 ±7.93	77.10 ±9.41	2.66 ±0.67	13.60 ±1.80
60	4	114.91 ±42.82	64.66 ±46.01	86.09 ±47.11	52.48 ±19.90	70.57 ±15.05	72.23 ±13.75	2.39 ±0.44	11.32 ±2.72
75	4	113.49 ±72.76	71.18 ±82.68	88.05 ±83.65	50.19 ±29.20	68.51 ±21.47	68.29 ±21.83	1.55 ±1.06	9.81 ±6.64

VCL-curvilinear velocity, VSL- straight-line velocity, VAP –average-path velocity, LIN -linearity, STR –straightness, WOB –wobble, ALH -amplitude of lateral head displacement and BCF –beat-cross frequency.

Table S3: Vervet sperm motility parameters over 120 minutes of exposure to $CuSO_4$ (0-250 µg/ml) (n=4)

Time (min)	$CuSO_4$ (µg/ml)	Total Motility (%)	Progressive Motility (%)	Rapid Spermatozoa (%)	Medium Spermatozoa (%)	Slow Spermatozoa (%)
	0	46,94 ± 24.54	23,22 ± 15,76 ^{a}	19,05 ± 14,05 ^{a}	10,14 ± 6,99	17,73 ± 5,22
	10	43,31 ± 20.71	21,46 ± 11,58 ^{a}	16,97 ± 5,73 ^{a}	6,52 ± 8,82	19,82 ± 7,20
15	50	42,65 ± 27.44	25,35 ± 25,95 ^{a}	20,06 ± 17,40 ^{a}	7,76 ± 10,38	14,83 ± 4,46
	100	32,89 ± 17.93	14,90 ± 9,16 ^{a}	12,07 ± 6,83 ^{a}	6,38 ± 3,84	14,45 ± 8,55
	250	7,31 ± 7.42	0,63 ± 0,76 ^{b}	0,48 ± 0,56 ^{b}	0,48 ± 0,97	6,35 ± 6,44
	0	40,82 ± 20.73 ^{a}	11,56 ± 7,24 ^{a}	9,15 ± 6,35 ^{a}	5,39 ± 3,75 ^{a}	26,29 ± 11,29
	10	37,64 ± 12.72 ^{a}	15,50 ± 3,94 ^{a}	11,66 ± 5,07 ^{ab}	5,89 ± 2,29 ^{a}	20,10 ± 12,20
60	50	31,72 ± 9.67 ^{a}	9,38 ± 6,91 ^{ab}	7,80 ± 6,78 ^{ac}	3,87 ± 0,42 ^{a}	20,05 ± 13,41
	100	14,55 ± 9.82 ^{b}	3,71 ± 3,27 ^{c}	2,58 ± 2,35 ^{d}	3,51 ± 2,89 ^{a}	8,46 ± 6,77
	250	0,38 ± 0.77 ^{b}	0,00 ± 0,00 ^{cd}	0,00 ± 0,00 ^{de}	0,00 ± 0,00 ^{b}	0,38 ± 0,77
	0	24,76 ± 12.96	5,88 ± 4,15 ^{a}	5,05 ± 3,57 ^{a}	1,22 ± 1,07	18,49 ± 9,49
	10	28,45 ± 11.66	7,80 ± 2,14 ^{ab}	5,85 ± 0,59 ^{a}	3,33 ± 1,48	19,27 ± 10,15
120	50	15,82 ± 5.72	3,86 ± 2,77 ^{ac}	2,32 ± 1,56 ^{ab}	2,79 ± 2,39	10,71 ± 3,00
	100	3,70 ± 7.41	0,31 ± 0,62 ^{d}	0,21 ± 0,41 ^{c}	0,21 ± 0,41	3,29 ± 6,58
	250	0,00 ± 0.00	0,00 ± 0,00 ^{d}	0,00 ± 0,00 ^{c}	0,00 ± 0,00	0,00 ± 0,00

$CuSO_4$-copper sulphate, a-e = values in columns with different superscript letters were significantly different among the concentrations of $CuSO_4$ ($P < 0.05$).

Table S4: Vervet sperm kinematic parameters over 120 minutes of exposure to $CuSO_4$ (0-250 µg/ml) (n=4)

Time (min)	$CuSO_4$ (µg/ml)	VCL (µm/s)	VSL (µm/s)	VAP (µm/s)	LIN (%)	STR (%)	WOB (%)	ALH (µm)	BCF (Hz)
	0	118,89 ± 37,94	56,71 ± 21,27	70,68 ± 24,37	47,22 ± 3,17	79,89 ± 3,76 ^{a}	59,08 ± 1,79 ^{a}	2,49 ± 1,02	17,00 ± 5,06
	10	125,18 ± 21,19	71,50 ± 13,60	81,68 ± 14,28	57,34 ± 6,68	87,42 ± 2,62 ^{b}	65,47 ± 5,87 ^{a}	3,08 ± 0,41	20,35 ± 4,46
15	50	121,29 ± 20,47	60,52 ± 12,88	74,03 ± 15,50	49,95 ± 7,95	81,70 ± 1,78 ^{ac}	61,03 ± 8,50 ^{a}	2,49 ± 0,56	20,81 ± 4,25
	100	94,94 ± 21,11	50,97 ± 22,48	61,58 ± 23,23	51,44 ± 15,54	80,40 ± 8,39 ^{abc}	62,95 ± 13,63 ^{a}	1,86 ± 0,62	18,78 ± 8,50
	250	31,72 ± 30,58	8,97 ± 9,26	14,24 ± 13,72	19,89 ± 14,30	44,00 ± 30,97 ^{d}	33,76 ± 22,60 ^{b}	0,55 ± 0,93	4,52 ± 6,84
	0	81,47 ± 14,41	36,76 ± 10,90	46,53 ± 11,22	44,76 ± 8,56	78,34 ± 5,27 ^{a}	56,80 ± 6,82 ^{a}	1,83 ± 0,76 ^{a}	14,17 ± 3,63 ^{a}
	10	100,64 ± 23,25	56,27 ± 28,95	67,86 ± 29,08	53,91 ± 16,41	80,77 ± 7,69 ^{ab}	65,73 ± 14,35 ^{a}	2,02 ± 0,43 ^{a}	14,81 ± 3,16 ^{a}
60	50	87,05 ± 34,19	44,33 ± 22,36	55,68 ± 23,81	49,08 ± 6,80	77,26 ± 9,47 ^{ac}	63,48 ± 2,61 ^{a}	1,52 ± 0,82 ^{a}	13,87 ± 5,94 ^{a}
	100	56,86 ± 39,73	20,51 ± 14,37	27,97 ± 19,31	27,10 ± 18,25	54,81 ± 36,66 ^{d}	37,06 ± 24,79 ^{b}	1,53 ± 1,16 ^{a}	13,44 ± 9,85 ^{a}
	250	3,46 ± 6,92	0,07 ± 0,15	0,22 ± 0,44	0,53 ± 1,06	8,33 ± 16,67 ^{e}	1,59 ± 3,19 ^{b}	0,00 ± 0,00 ^{b}	0,00 ± 0,00 ^{b}

	0	71,96 ± 11,96	37,56 ± 16,56	46,64 ± 14,57	51,03 ± 18,24 ^{a}	78,13 ± 10,56 ^{a}	63,98 ± 13,62	1,68 ± 0,91	15,67 ± 4,42 ^{a}
	10	80,94 ± 6,45	42,58 ± 8,53	52,28 ± 4,51	53,12 ± 12,69 ^{a}	81,03 ± 12,57 ^{a}	64,86 ± 6,96	1,47 ± 0,70	14,40 ± 3,62 ^{a}
120	50	61,57 ± 28,75	25,20 ± 20,39	34,69 ± 22,57	34,91 ± 20,07 ^{a}	64,43 ± 20,79 ^{a}	50,56 ± 17,00	1,21 ± 1,06	10,79 ± 8,26 ^{a}
	100	12,59 ± 25,18	2,06 ± 4,12	4,14 ± 8,28	4,10 ± 8,19 ^{b}	12,46 ± 24,91 ^{b}	8,22 ± 16,44	0,13 ± 0,26	1,40 ± 2,80 ^{b}
	250	0,00 ± 0,00	0,00 ± 0,00	0,00 ± 0,00	0,00 ± 0,00 ^{b}	0,00 ± 0,00 ^{b}	0,00 ± 0,00	0,00 ± 0,00	0,00 ± 0,00 ^{b}

$CuSO_4$-copper sulphate, VCL-curvilinear velocity, VSL-straight-line velocity, VAP-average-path velocity, LIN-linearity, STR-straightness, WOB-wobble, ALH-amplitude of lateral head displacement and BCF-beat-cross frequency, a-e = values in columns with different superscript letters were significantly different among the concentrations of $CuSO_4$ ($P < 0.05$).

Table S5: Vervet sperm motility parameters over 120 minutes of exposure to $CdCl_2$ (0-500 μg/ml) (n=4)

Time (min)	$CdCl_2$ (μg/ml)	Total Motility (%)	Progressive Motility (%)	Rapid Spermatozoa (%)	Medium Spermatozoa (%)	Slow Spermatozoa (%)
	0	45,70 ± 35,36	26,76 ± 31,64	23,19 ± 31,14	6,29 ± 3,53	16,22 ± 3,61
	10	42,61 ± 36,69	23,87 ± 32,81	21,76 ± 32,30	3,92 ± 2,31	16,94 ± 5,89
15	50	41,35 ± 33,80	19,59 ± 31,35	17,56 ± 30,32	4,34 ± 3,22	19,45 ± 3,35
	100	29,57 ± 43,88	17,76 ± 34,09	16,62 ± 32,27	3,50 ± 5,86	9,46 ± 6,66
	500	1,21 ± 2,41	0,00 ± 0,00	0,00 ± 0,00	0,00 ± 0,00	1,21 ± 2,41
	0	39,72 ± 40,6	23,75 ± 31,15 ^{a}	21,52 ± 30,21 ^{a}	3,68 ± 3,15	14,52 ± 8,74 ^{a}
	10	39,76 ± 35,3	19,13 ± 29,22 ^{a}	16,96 ± 27,39 ^{a}	4,36 ± 4,07	18,44 ± 5,77 ^{a}
60	50	21,99 ± 33,6	8,22 ± 15,52 ^{a}	7,10 ± 13,62 ^{a}	2,30 ± 3,27	12,58 ± 16,94 ^{a}
	100	14,74 ± 28,60	3,04 ± 6,07 ^{b}	2,09 ± 4,18 ^{b}	1,94 ± 3,65	10,71 ± 20,73 ^{b}
	500	0,00 ± 0,00	0,00 ±0,00 ^{c}	0,00 ± 0,00 ^{c}	0,00 ± 0,00	0,00 ± 0,00 ^{c}
	0	32,82 ± 44,20 ^{a}	16,50 ± 27,81 ^{a}	14,78 ± 26,39 ^{a}	5,93 ± 7,59 ^{a}	12,11 ± 11,80
	10	26,71 ± 34,90 ^{a}	11,36 ± 21,63 ^{a}	9,55 ± 18,41 ^{a}	3,24 ± 6,10 ^{a}	13,91 ± 10,73
120	50	10,40 ± 13,50 ^{a}	0,36 ± 0,42 ^{b}	0,10 ± 0,12 ^{b}	0,82 ± 1,01 ^{b}	9,48 ± 12,79
	100	6,82 ± 13,60 ^{a}	0,28 ± 0,56 ^{b}	0,14 ± 0,28 ^{b}	0,97 ± 1,95 ^{b}	5,71 ± 11,41
	500	0,00 ± 0,00 ^{b}	0,00 ± 0,00 ^{b}	0,00 ± 0,00 ^{b}	0,00 ± 0,00 ^{b}	0,00 ± 0,00

$CdCl_2$-cadmium chloride, a–c = values in columns with different superscript letters were significantly different among the concentrations of $CdCl_2$ ($P<0.05$).

Table S6: Vervet sperm kinematic parameters over 120 minutes of exposure to $CdCl_2$ (0-500 μg/ml) (n=4)

Time (min)	$CdCl_2$ (μg/ml)	VCL (μm/s)	VSL (μm/s)	VAP (μm/s)	LIN (%)	STR (%)	WOB (%)	ALH (μm)	BCF (Hz)
	0	112,17 ± 56,84	63,95 ± 39,75	79,09 ± 50,72	54,27 ± 12,39	80,08 ± 9,92	67,34 ± 10,84	2,49 ± 0,54 $_{a}$	15,66 ± 3,30 ^{a}
	10	105,76 ± 59,64	59,17 ± 32,56	74,14 ± 45,72	57,16 ± 15,32	81,40 ± 7,29	69,65 ± 13,29	2,26 ± 1,14 $_{a}$	14,52 ± 4,07 ^{a}
15	50	93,44 ± 50,15	52,32 ± 31,98	66,41 ± 42,45	54,73 ± 11,88	79,17 ± 6,65	68,73 ± 10,48	2,38 ± 0,65 $_{a}$	15,27 ± 1,85 ^{a}
	100	78,99 ± 67,74	37,21 ± 44,13	50,32 ± 56,24	37,00 ± 19,96	65,67 ± 15,35	52,62 ± 21,95	1,33 ± 1,36 $_{b}$	7,81 ± 7,01 ^{b}
	500	9,20 ± 18,40	2,86 ± 5,72	5,35 ± 10,70	7,77 ± 15,54	13,36 ± 26,71	14,54 ± 29,08	0,00 ± 0,00 $_{c}$	0,00 ± 0,00 ^{c}
	0	103,95 ± 50,99	59,55 ± 38,91	73,53 ± 47,69	52,24 ± 25,09 ^{a}	75,36 ± 20,41 $_{a}$	65,38 ± 21,72 $_{a}$	1,80 ± 0,99 $_{a}$	10,70 ± 8,50 ^{a}
	10	91,84 ± 48,55	52,76 ± 36,26	64,52 ± 45,21	54,96 ± 12,87 ^{a}	82,07 ± 4,13 $_{a}$	66,75 ± 14,55 $_{a}$	1,84 ± 0,75 $_{a}$	13,54 ± 3,94 ^{a}
60	50	52,15 ± 43,61	28,23 ± 32,77	39,62 ± 36,04	35,66 ± 30,47 ^{a}	48,20 ± 40,07 $_{a}$	55,59 ± 37,84 $_{a}$	0,94 ± 1,10 $_{a}$	6,90 ± 7,96 ^{a}
	100	32,42 ± 37,57	15,38 ± 18,01	20,49 ± 23,90	23,64 ± 27,39 ^{b}	37,47 ± 43,28 $_{b}$	31,52 ± 36,46 $_{b}$	0,46 ± 0,93 $_{b}$	3,17 ± 6,35 ^{b}
	500	0,00 ± 0,00	0,00 ± 0,00	0,00 ± 0,00	0,00 ± 0,00 ^{c}	0,00 ± 0,00 $_{c}$	0,00 ± 0,00 $_{c}$	0,00 ± 0,00 $_{c}$	0,00 ± 0,00 ^{c}
	0	81,28 ± 63,87	46,71 ± 38,26 ^{a}	56,68 ± 46,95 ^{a}	43,44 ± 34,13	62,17 ± 42,36 $_{a}$	51,60 ± 37,58	1,92 ± 1,49 $_{a}$	13,42 ± 9,59 ^{a}
	10	63,77 ± 43,87	28,76 ± 34,87 ^{a}	38,07 ± 42,15 ^{a}	35,68 ± 19,15	69,70 ± 10,96 $_{a}$	49,49 ± 20,47	0,98 ± 0,94 $_{a}$	8,39 ± 5,59 ^{a}
120	50	25,21 ± 29,28	6,67 ± 7,81 ^{b}	11,69 ± 13,52 ^{a}	13,17 ± 15,24	28,41 ± 33,04 $_{b}$	23,26 ± 26,90	1,28 ± 1,49 $_{a}$	3,92 ± 5,43 ^{b}
	100	13,47 ± 26,94	3,22 ± 6,43 ^{c}	6,61 ± 13,23 ^{b}	5,97 ± 11,94	12,16 ± 24,32 $_{c}$	12,27 ± 24,55	0,81 ± 1,63 $_{b}$	0,99 ± 1,99 ^{c}
	500	0,00 ± 0,00	0,00 ± 0,00 ^{c}	0,00 ± 0,00 ^{b}	0,00 ± 0,00	0,00 ± 0,00 $_{c}$	0,00 ± 0,00	0,00 ± 0,00 $_{c}$	0,00 ± 0,00 ^{c}

$CdCl_2$-cadmium chloride, VCL-curvilinear velocity, VSL-straight-line velocity, VAP-average-path velocity, LIN-linearity, STR-straightness, WOB-wobble, ALH-amplitude of lateral head displacement and BCF-beat-cross frequency, a-c = values in columns with different superscript letters were significantly different among the concentrations of $CdCl_2$ ($P<0.05$).

Table S7: Baboon sperm motility parameters over 75 minutes of exposure to $CuSO_4$ (0-250 μg/ml) (n=4)

Time (mins)	$CuSO_4$ (μg/ml)	Total Motility (%)	Progressive Motility (%)	Rapid Spermatozoa (%)	Medium Spermatozoa (%)	Slow Spermatozoa (%)
	0	61.95 ± 13.14	35.98 ± 9.16	0.83 ± 0.86	13.38 ± 5.90	33.54 ± 9.33
	10	60.37 ± 18.75	29.34 ± 9.64	1.78 ± 2.51	11.22 ± 5.84	29.96 ± 7.52
15	50	50.53 ± 32.91	22.68 ± 16.22	1.49 ± 2.79	8.29 ± 8.17	25.57 ± 18.01
	100	62.68 ± 17.21	29.74 ± 13.62	1.95 ± 2.69	11.20 ± 9.41	31.50 ± 4.97
	250	54.94 ± 14.92	26.63 ± 9.97	1.29 ± 1.77	9.00 ± 5.60	28.99 ± 9.12
	0	50.72 ± 12.50	16.39 ± 7.86	0.11 ± 0.22	3.45 ± 4.01	25.93 ± 3.52
	10	45.02 ± 26.63	13.04 ± 13.83	0.82 ± 1.63	3.96 ± 6.15	17.91 ± 10.21
45	50	37.94 ± 29.16	10.08 ± 11.18	0.31 ± 0.62	2.01 ± 4.01	17.45 ± 12.93
	100	50.71 ± 15.48	14.38 ± 16.25	0.97 ± 1.94	3.64 ± 6.29	22.39 ± 7.39
	250	25.43 ± 20.85	9.45 ± 10.81	0.43 ± 0.87	2.91 ± 4.59	11.63 ± 11.02
	0	54.52 ± 13.89	15.09 ± 13.46	0.09 ± 0.16 [a]	3.25 ± 4.78	24.72 ± 11.62
	10	44.03 ± 27.94	12.92 ± 16.27	1.01 ± 1.76 [a]	3.85 ± 5.80	17.61 ± 11.45
75	50	34.16 ± 29.16	9.86 ± 13.31	0.15 ± 0.26 [a]	2.74 ± 4.75	13.98 ± 12.91
	100	47.06 ± 9.28	12.20 ± 12.46	0.65 ± 1.13 [a]	3.50 ± 5.59	18.44 ± 8.57
	250	16.83 ± 15.58	4.37 ± 3.79	0.00 ± 0.00 [b]	0.72 ± 0.64	8.20 ± 7.63

$CuSO_4$-copper sulphate, a ,b = values in columns with different superscript letters were significantly different among the concentrations of $CuSO_4$ ($P < 0.05$).

Table S8: Baboon sperm kinematic parameters over 75 minutes of exposure to $CuSO_4$ (0-250 µg/ml) (n=4)

Time (min)	$CuSO_4$ (µg/ml)	VCL (µm/s)	VSL (µm/s)	VAP (µm/s)	LIN (%)	STR (%)	WOB (%)	ALH (µm)	BCF (Hz)
	0	116.56 ± 18.75	88.07 ± 18.44	101.54 ± 21.38	75.21 ± 5.08 ^{a}	86.78 ± 1.23 ^{a}	86.68 ± 5.85	1.81 ± 0.15	15.42 ± 0.82 ^{a}
	10	110.46 ± 17.69	70.68 ± 21.12	83.40 ± 22.65	64.18 ± 16.23 ^{a}	84.21 ± 4.10 ^{a}	75.64 ± 16.15	2.26 ± 0.68	15.95 ± 3.45 ^{a}
15	50	94.30 ± 35.28	59.82 ± 38.34	73.53 ± 42.22	57.35 ± 28.41 ^{b}	74.02 ± 19.82 ^{b}	72.33 ± 25.89	1.42 ± 0.99	11.20 ± 7.60 ^{b}
	100	106.22 ± 31.29	76.17 ± 26.41	90.14 ± 30.18	70.96 ± 4.43 ^{ab}	84.33 ± 1.57 ^{ab}	84.14 ± 5.16	1.86 ± 0.24	14.35 ± 0.72 ^{ab}
	250	106.36 ± 15.57	75.78 ± 17.84	89.13 ± 18.40	70.59 ± 9.22 ^{ab}	84.54 ± 4.69 ^{ab}	83.26 ± 7.26	1.82 ± 0.30	14.16 ± 0.49 ^{ab}
	0	80.56 ± 16.30	53.76 ± 10.81	63.03 ± 13.48	66.98 ± 6.05	85.51 ± 2.74 ^{a}	78.25 ± 5.13	1.74 ± 0.24	15.98 ± 1.57
	10	79.68 ± 31.92	46.14 ± 32.86	55.90 ± 35.20	54.83 ± 22.53	78.78 ± 12.12 ^{a}	67.30 ± 20.14	1.69 ± 0.60	14.73 ± 5.28
45	50	63.98 ± 35.76	39.99 ± 33.59	46.86 ± 35.77	52.80 ± 25.14	78.38 ± 13.57 ^{a}	64.35 ± 23.46	1.09 ± 0.82	9.92 ± 7.41
	100	77.05 ± 30.98	49.01 ± 28.54	57.91 ± 30.69	60.84 ± 9.60	83.20 ± 4.27 ^{a}	72.85 ± 7.86	1.48 ± 0.59	13.72 ± 4.01
	250	60.84 ± 46.61	42.00 ± 32.34	48.30 ± 37.49	51.85 ± 34.69	65.41 ± 43.61 ^{b}	59.45 ± 39.76	1.18 ± 0.90	11.37 ± 7.95
	0	74.75 ± 22.90	47.60 ± 16.07	56.43 ± 20.17	63.36 ± 2.70 ^{a}	84.76 ± 1.70 ^{a}	74.80 ± 4.36 ^{a}	1.68 ± 0.19	16.86 ± 1.22 ^{a}
	10	81.58 ± 34.20	46.41 ± 37.15	56.01 ± 40.70	52.69 ± 25.06 ^{a}	78.96 ± 11.94 ^{a}	64.39 ± 24.04 ^{a}	1.86 ± 0.32	17.78 ± 6.20 ^{a}
75	50	53.64 ± 53.20	35.14 ± 36.71	41.04 ± 42.75	42.60 ± 37.23 ^{b}	56.98 ± 49.35 ^{b}	49.83 ± 43.50 ^{b}	1.10 ± 1.01	10.20 ± 8.93 ^{b}
	100	77.73 ± 33.12	48.40 ± 28.03	58.19 ± 33.65	59.38 ± 11.37 ^{ab}	83.07 ± 0.72 ^{ab}	71.42 ± 13.31 ^{ab}	1.55 ± 0.53	12.23 ± 3.88 ^{c}
	250	50.25 ± 43.76	33.54 ± 29.87	38.79 ± 34.22	44.28 ± 38.70 ^{bc}	57.47 ± 49.82 ^{bc}	51.29 ± 44.60 ^{bc}	1.14 ± 0.99	11.34 ± 9.82 ^{c}

$CuSO_4$-copper sulphate, VCL-curvilinear velocity, VSL- straight-line velocity, VAP-average-path velocity, LIN-linearity, STR-straightness, WOB-wobble, ALH-amplitude of lateral head displacement and BCF-beat-cross frequency, a-c = values in columns with different superscript letters were significantly different among the concentrations of $CuSO_4$ ($P < 0.05$).

Table S9: Baboon sperm motility parameters over 75 minutes of exposure to $CdCl_2$ (0-500 µg/ml) (n=4)

Time (min)	$CdCl_2$ (µg/ml)	Total Motility (%)	Progressive Motility (%)	Rapid Spermatozoa (%)	Medium Spermatozoa (%)	Slow Spermatozoa (%)
	0	66.88 ± 34.88	46.11 ± 30.47	1.07 ± 1.96	18.26 ± 16.32	40.22 ± 19.00
	10	74.02 ± 36.83	51.66 ± 30.14	5.11 ± 6.35	25.33 ± 16.46	32.48 ± 21.58
15	50	65.08 ± 43.81	47.70 ± 27.56	4.86 ± 7.91	19.22 ± 13.37	34.61 ± 20.48
	100	69.55 ± 36.04	45.30 ± 30.93	6.35 ± 8.94	16.96 ± 15.38	34.34 ± 22.64
	500	52.26 ± 39.17	24.80 ± 23.57	0.83 ± 0.95	10.05 ± 11.65	25.58 ± 21.73
	0	57.10 ± 34.14	26.15 ± 19.33	0.15 ± 0.18 ^{a}	7.17 ± 6.25	34.10 ± 22.53
	10	64.53 ± 29.44	36.79 ± 22.74	2.14 ± 1.74 ^{b}	8.76 ± 11.72	42.01 ± 23.46
45	50	61.95 ± 33.13	35.87 ± 20.84	2.44 ± 2.82 ^{c}	11.67 ± 10.07	36.40 ± 24.07

	100	61.78 ± 27.10	31.84 ± 19.66	2.19 ± 2.45 ^{c}	11.38 ± 12.74	30.93 ± 18.77
	500	0.90 ± 1.08	0.00 ± 0.00	0.00 ± 0.00 ^{d}	0.00 ± 0.00	0.09 ± 0.18
	0	73.21 ± 16.45 ^{a}	32.19 ± 13.78	0.00 ± 0.00 ^{a}	6.68 ± 5.40 ^{a}	49.02 ± 16.60 ^{a}
	10	79.20 ± 7.32 ^{a}	34.09 ± 11.53	1.13 ± 1.67 ^{b}	6.56 ± 6.48 ^{a}	48.40 ± 8.93 ^{a}
75	50	73.58 ± 7.40 ^{a}	29.06 ± 21.53	1.84 ± 2.72 ^{b}	8.40 ± 10.91 ^{b}	38.03 ± 6.85 ^{a}
	100	67.76 ± 8.30 ^{a}	22.74 ± 13.87	0.00 ± 0.00 ^{ac}	6.89 ± 8.04 ^{ab}	33.39 ± 8.93 ^{a}
	500	0.00 ± 0.00 ^{b}	0.00 ± 0.00	0.00 ± 0.00 ^{ac}	0.00 ± 0.00 ^{c}	0.00 ± 0.00 ^{b}

$CdCl_2$-cadmium chloride, a–c = values in columns with different superscript letters were significantly different among the concentrations of $CdCl_2$ (P<0.05).

Table S10: Baboon sperm kinematic parameters over 75 minutes of exposure to $CdCl_2$ (0-500 μg/ml) (n=4)

Time (min)	$CdCl_2$ (μg/ml)	VCL (μm/s)	VSL (μm/s)	VAP (μm/s)	LIN (%)	STR (%)	WOB (%)	ALH (μm)	BCF (Hz)
	0	125.47 ± 22.64	91.47 ± 15.29	108.71 ± 22.11	73.11 ± 3.89 ^{a}	84.60 ± 4.13 ^{a}	86.43 ± 2.59 ^{a}	2.01 ± 0.38	16.48 ± 2.92
	10	155.71 ± 34.36	95.45 ± 28.29	138.03 ± 36.94	62.04 ± 14.06 ^{b}	71.26 ± 18.53 ^{a}	87.97 ± 5.61 ^{a}	1.87 ± 1.08	11.78 ± 4.90
15	50	137.41 ± 36.18	105.28 ± 29.69	121.27 ± 35.81	76.36 ± 6.28 ^{c}	87.02 ± 4.16 ^{a}	87.69 ± 4.10 ^{a}	1.81 ± 0.58	12.70 ± 3.79
	100	145.31 ± 45.38	113.72 ± 44.78	129.72 ± 48.40	76.77 ± 9.57 ^{ab}	87.11 ± 5.53 ^{a}	87.93 ± 6.34 ^{a}	1.85 ± 0.30	15.64 ± 3.02
	500	76.33 ± 53.98	50.87 ± 35.89	60.84 ± 44.04	50.01 ± 33.39 ^{d}	63.23 ± 42.29 ^{b}	59.40 ± 39.72 ^{b}	1.42 ± 0.97	11.69 ± 7.88
	0	101.48 ± 19.37	71.66 ± 21.87 ^{a}	85.58 ± 22.93	69.49 ± 8.87	83.15 ± 4.23 ^{a}	83.38 ± 7.10 ^{a}	1.82 ± 0.55	12.64 ± 3.71
	10	113.92 ± 28.02	81.13 ± 24.60 ^{a}	94.82 ± 29.41	70.50 ± 4.06	85.64 ± 1.70 ^{a}	82.34 ± 5.02 ^{a}	1.79 ± 0.29	15.17 ± 1.48
45	50	124.39 ± 29.22	98.39 ± 31.25 ^{a}	111.94 ± 33.59	77.93 ± 7.53	87.52 ± 1.93 ^{a}	88.94 ± 6.68 ^{a}	1.79 ± 0.25	13.95 ± 1.86
	100	112.21 ± 29.68	85.12 ± 32.26 ^{a}	96.12 ± 33.22	74.14 ± 9.76	87.72 ± 4.43 ^{a}	84.32 ± 7.75 ^{a}	1.86 ± 0.31	15.22 ± 2.05
	500	22.59 ± 26.34	6.15 ± 8.19 ^{b}	9.05 ± 11.26	14.29 ± 20.01	32.70 ± 38.47 ^{b}	20.78 ± 27.10 ^{b}	0.00 ± 0.00	0.00 ± 0.00
	0	92.15 ± 15.45 ^{a}	63.41 ± 13.05 ^{a}	76.21 ± 15.88 ^{a}	68.53 ± 3.17 ^{a}	83.23 ± 0.72	82.35 ± 4.38	2.00 ± 0.22	13.75 ± 0.62
	10	93.02 ± 19.14 ^{a}	62.36 ± 17.30 ^{a}	75.08 ± 20.57 ^{a}	66.55 ± 6.95 ^{a}	83.00 ± 0.83	80.14 ± 7.69	1.87 ± 0.19	14.76 ± 1.29
75	50	93.30 ± 36.77 ^{a}	63.65 ± 34.71 ^{a}	75.54 ± 39.38 ^{a}	65.82 ± 9.46 ^{a}	83.66 ± 2.11	78.58 ± 9.87	1.81 ± 0.11	14.96 ± 0.72
	100	81.72 ± 20.22 ^{a}	54.97 ± 19.92 ^{a}	64.77 ± 22.27 ^{a}	66.20 ± 10.83 ^{a}	84.46 ± 2.29	78.19 ± 10.87	1.84 ± 0.14	14.77 ± 1.36
	500	0.00 ± 0.00 ^{b}	0.00 ± 0.00 ^{b}	0.00 ± 0.00 ^{b}	0.00 ± 0.00 ^{b}	0.00 ± 0.00	0.00 ± 0.00	0.00 ± 0.00	0.00 ± 0.00

$CdCl_2$-cadmium chloride, VCL-curvilinear velocity, VSL-straight-line velocity, VAP-average-path velocity, LIN-linearity, STR-straightness, WOB-wobble, ALH-amplitude of lateral head displacement and BCF-beat-cross frequency, a-d = values in columns with different superscript letters were significantly different among the concentrations of $CdCl_2$ (P<0.05).

Table S11: Rhesus sperm motility parameters over 120 minutes of exposure to $CuSO_4$ (0-250 μg/ml) (n=3)

Time (min)	$CuSO_4$ (μg/ml)	Total Motility (%)	Progressive Motility (%)	Rapid Spermatozoa (%)	Medium Spermatozoa (%)	Slow Spermatozoa (%)
	0	64.72 ± 26.00	51.14 ± 24.64	44.68 ± 25.92	9.87 ± 6.84	10.18 ± 1.45
	10	69.16 ± 23.94	58.15 ± 25.00	53.18 ± 27.74	8.33 ± 8.90	7.65 ± 5.09
15	50	66.69 ± 23.93	54.20 ± 20.72	47.64 ± 23.30	9.18 ± 8.00	9.87 ± 2.13
	100	73.85 ± 22.12	59.09 ± 20.72	52.07 ± 24.64	10.63 ± 7.78	11.14 ± 4.59
	250	52.15 ± 43.93	41.26 ± 38.46	35.75 ± 37.57	8.25 ± 8.13	8.15 ± 4.67
	0	57.35 ± 24.58	33.01 ± 13.71	25.95 ± 11.37	12.02 ± 9.17	19.37 ± 10.99
	10	58.51 ± 23.63	34.65 ± 10.45	24.77 ± 9.47	13.86 ± 8.05	19.87 ± 12.22
60	50	55.66 ± 26.45	30.44 ± 15.12	20.11 ± 11.51	14.30 ± 10.40	21.25 ± 12.88
	100	49.07 ± 36.85	23.14 ± 22.96	13.87 ± 15.68	12.65 ± 9.78	22.54 ± 13.41
	250	4.55 ± 5.06	1.01 ± 1.75	0.51 ± 0.87	0.71 ± 1.22	3.34 ± 3.19
	0	35.03 ± 37.50	11.58 ± 12.48 ^{a}	4.70 ± 5.17	11.02 ± 13.70 ^{a}	19.32 ± 19.13
	10	34.11 ± 29.58	9.55 ± 6.12 ^{a}	3.12 ± 1.66	9.24 ± 9.32 ^{a}	21.75 ± 20.33
120	50	36.43 ± 34.80	10.62 ± 10.54 ^{a}	3.95 ± 3.69	11.86 ± 14.30 ^{a}	20.62 ± 17.69
	100	27.74 ± 30.96	4.05 ± 4.19 ^{b}	1.06 ± 1.28	5.01 ± 4.09 ^{b}	21.67 ± 26.07
	250	0.18 ± 0.32	0.18 ± 0.32 ^{c}	0.18 ± 0.32	0.00 ± 0.00 ^{c}	0.00 ± 0.00

$CuSO_4$-copper sulphate, a-c = values in columns with different superscript letters were significantly different among the concentrations of $CuSO_4$ ($P < 0.05$).

Table S12: Rhesus sperm kinematic parameters over 120 minutes of exposure to $CuSO_4$ (0-250 μg/ml) (n=3)

Time (min)	$CuSO_4$ (μg/ml)	VCL (μm/s)	VSL (μm/s)	VAP (μm/s)	LIN (%)	STR (%)	WOB (%)	ALH (μm)	BCF (Hz)
	0	176.69 ± 40.47	139.14 ± 41.15	154.49 ± 42.51	77.79 ± 6.29	89.63 ± 2.76	86.71 ± 4.91	2.57 ± 0.21	16.90 ± 1.18 ^{a}
	10	204.26 ± 59.85	167.38 ± 60.76	182.39 ± 59.82	80.56 ± 8.05	90.83 ± 4.29	88.56 ± 5.13	2.53 ± 0.42	17.19 ± 0.41 ^{a}
15	50	189.00 ± 40.35	155.56 ± 43.00	171.77 ± 44.96	81.49 ± 6.29	90.34 ± 2.94	90.15 ± 5.16	2.16 ± 0.04	16.14 ± 1.33 ^{a}
	100	168.23 ± 41.03	126.06 ± 35.80	141.86 ± 40.63	74.39 ± 4.37	88.87 ± 1.30	83.69 ± 4.37	2.59 ± 0.31	17.92 ± 0.47 ^{a}
	250	149.88 ± 47.20	119.65 ± 38.51	134.07 ± 45.17	80.06 ± 10.76	89.49 ± 4.77	89.24 ± 7.68	2.12 ± 0.31	15.47 ± 2.92 ^{b}
	0	133.74 ± 50.35	99.09 ± 63.22	110.62 ± 61.66	70.07 ± 18.6	87.20 ± 6.73 ^{a}	79.82 ± 15.93 ^{a}	2.07 ± 0.68 ^{a}	18.48 ± 2.85 ^{a}
	10	129.99 ± 44.89	99.79 ± 54.07	108.87 ± 51.81	73.76 ± 14.22	89.94 ± 5.44 ^{a}	81.59 ± 10.81 ^{a}	2.17 ± 0.38 ^{a}	17.45 ± 1.60 ^{a}

60	50	122.76 ± 43.02	92.45 ± 54.69	101.43 ± 51.71	71.50 ± 16.72	88.79 ± 7.02 ^{a}	79.93 ± 12.29 ^{a}	2.27 ± 0.43 ^{a}	17.50 ± 1.99 ^{a}
	100	93.63 ± 12.68	68.21 ± 17.47	78.95 ± 13.69	72.13 ± 11.53	85.48 ± 8.49 ^{a}	84.05 ± 5.43 ^{a}	2.01 ± 0.27 ^{a}	16.20 ± 0.42 ^{a}
	250	38.58 ± 44.15	19.80 ± 32.52	25.75 ± 38.80	24.42 ± 36.28	37.20 ± 41.19 ^{b}	34.95 ± 41.72 ^{b}	0.72 ± 1.25 ^{b}	4.68 ± 8.11 ^{b}
	0	85.03 ± 13.14	48.17 ± 22.66 ^{a}	56.36 ± 22.25 ^{a}	55.05 ± 17.13 ^{a}	83.97 ± 5.76 ^{a}	64.87 ± 15.50 ^{a}	1.98 ± 0.45 ^{a}	16.73 ± 5.70 ^{a}
	10	90.65 ± 31.00	55.72 ± 36.12 ^{a}	63.52 ± 35.12 ^{a}	57.66 ± 17.38 ^{a}	84.96 ± 7.63 ^{a}	67.11 ± 14.42 ^{a}	2.15 ± 0.23 ^{a}	16.65 ± 3.12 ^{a}
120	50	85.51 ± 18.33	52.94 ± 35.03 ^{a}	60.11 ± 35.52 ^{b}	58.36 ± 25.70 ^{a}	84.62 ± 9.72 ^{a}	67.40 ± 21.46 ^{a}	2.26 ± 0.19 ^{a}	17.51 ± 3.66 ^{a}
	100	56.87 ± 15.69	25.38 ± 4.99 ^{b}	37.79 ± 2.32 ^{c}	45.40 ± 4.56 ^{a}	67.16 ± 12.86 ^{b}	70.01 ± 20.09 ^{a}	1.29 ± 1.18 ^{a}	11.29 ± 9.93 ^{a}
	250	14.13 ± 24.47	2.00 ± 3.47 ^{b}	3.93 ± 6.80 ^{d}	4.73 ± 8.19 ^{b}	17.02 ± 29.47 ^{b}	9.26 ± 16.04 ^{b}	0.00 ± 0.00 ^{b}	0.00 ± 0.00 ^{b}

$CuSO_4$-copper sulphate, VCL-curvilinear velocity, VSL- straight-line velocity, VAP-average-path velocity, LIN-linearity, STR-straightness, WOB-wobble, ALH-amplitude of lateral head displacement and BCF-beat-cross frequency, a-d = values in columns with different superscript letters were significantly different among the concentrations of $CuSO_4$ ($P < 0.05$).

Table S13: Rhesus sperm motility parameters over 120 minutes of exposure to $CdCl_2$ (0-500 μg/ml) (n=3)

Time (min)	$CdCl_2$ (μg/ml)	Total Motility (%)	Progressive Motility (%)	Rapid Spermatozoa (%)	Medium Spermatozoa (%)	Slow Spermatozoa (%)
	0	62.33 ± 26.27	47.88 ± 23.30	42.48 ± 24.70	9.02 ± 2.47 ^{a}	10.83 ± 3.06
	10	59.29 ± 31.35	46.01 ± 30.48	39.68 ± 30.76	10.83 ± 1.13 ^{a}	8.78 ± 3.58
15	50	63.90 ± 32.66	50.43 ± 33.31	45.17 ± 34.89	8.41 ± 2.23 ^{a}	10.31 ± 2.78
	100	63.63 ± 30.00	45.36 ± 29.67	40.38 ± 30.70	8.61 ± 1.21 ^{a}	14.64 ± 4.23
	500	43.83 ± 38.44	24.61 ± 31.16	18.66 ± 27.81	8.07 ± 5.91 ^{b}	17.10 ± 7.18
	0	43.25 ± 45.46	27.48 ± 30.27	22.76 ± 25.42	7.20 ± 7.12	13.29 ± 13.11
	10	55.00 ± 26.83	31.96 ± 25.17	23.33 ± 20.88	13.83 ± 5.85	17.84 ± 2.00
60	50	46.14 ± 37.55	30.95 ± 34.56	25.00 ± 31.61	9.01 ± 3.76	12.14 ± 2.63
	100	49.25 ± 36.79	30.46 ± 30.63	23.21 ± 29.03	12.38 ± 4.67	13.66 ± 3.58
	500	1.83 ± 1.58	0.09 ± 0.16	0.09 ± 0.16	0.09 ± 0.16	1.65 ± 1.45
	0	49.06 ± 45.88 ^{a}	27.93 ± 35.51 ^{a}	19.18 ± 25.53 ^{a}	12.11 ± 10.75 ^{a}	17.78 ± 9.61 ^{a}
	10	45.16 ± 47.53 ^{a}	29.33 ± 39.49 ^{a}	24.21 ± 33.05 ^{a}	8.11 ± 8.68 ^{a}	12.83 ± 5.80 ^{a}
120	50	45.61 ± 54.72 ^{a}	28.58 ± 40.08 ^{a}	21.94 ± 31.03 ^{a}	8.88 ± 11.89 ^{a}	14.79 ± 11.80 ^{a}
	100	41.74 ± 56.56 ^{a}	18.59 ± 25.67 ^{a}	12.28 ± 17.06 ^{a}	10.76 ± 14.60 ^{a}	18.70 ± 24.90 ^{a}
	500	0.81 ± 1.15 ^{b}	0.00 ± 0.00 ^{b}	0.00 ± 0.00 ^{b}	0.00 ± 0.00 ^{b}	0.81 ± 1.15 ^{b}

$CdCl_2$-cadmium chloride, a, b = values in columns with different superscript letters were significantly different among the concentrations of $CdCl_2$ ($P<0.05$).

Table S14: Rhesus sperm kinematic parameters over 120 minutes of exposure to $CdCl_2$ (0-500 μg/ml) (n=3)

Time (min)	$CdCl_2$ (μg/ml)	VCL (μm/s)	VSL (μm/s)	VAP (μm/s)	LIN (%)	STR (%)	WOB (%)	ALH (μm)	BCF (Hz)
	0	166.46 ± 35.79	117.58 ± 14.08	137.76 ± 27.69	71.58 ± 6.70 ^{a}	86.24 ± 6.65	82.94 ± 1.66 ^{a}	2.53 ± 0.24	16.28 ± 1.04
	10	163.08 ± 59.12	115.21 ± 43.59	134.87 ± 54.96	70.32 ± 1.58 ^{a}	86.06 ± 2.67	81.78 ± 3.83 ^{a}	2.60 ± 0.29	16.03 ± 1.04
15	50	172.98 ± 67.00	126.64 ± 48.34	146.25 ± 61.87	73.16 ± 4.07 ^{a}	87.63 ± 4.97	83.56 ± 4.06 ^{a}	2.56 ± 0.31	16.13 ± 1.10
	100	152.31 ± 55.11	109.12 ± 40.50	125.84 ± 49.90	71.40 ± 1.28 ^{a}	87.34 ± 3.29	81.83 ± 3.58 ^{a}	2.60 ± 0.33	16.31 ± 1.69
	500	103.61 ± 47.94	69.91 ± 37.56	82.27 ± 45.12	65.35 ± 11.37 ^{b}	84.83 ± 5.00	76.80 ± 10.39 ^{b}	2.16 ± 0.23	15.92 ± 0.48
	0	89.95 ± 78.89	64.99 ± 56.96 ^{a}	75.56 ± 66.95	48.18 ± 41.73 ^{a}	57.59 ± 49.97 ^{a}	55.83 ± 48.43 ^{a}	1.44 ± 1.24 ^{a}	11.44 ± 9.97 ^{a}
	10	117.88 ± 21.12	78.35 ± 20.54 ^{a}	92.05 ± 23.19	65.83 ± 5.55 ^{a}	84.96 ± 2.82 ^{b}	77.45 ± 5.35 ^{a}	2.41 ± 0.19 ^{a}	15.77 ± 0.95 ^{a}
60	50	118.89 ± 45.37	76.95 ± 43.22 ^{a}	91.46 ± 48.02	61.94 ± 10.92 ^{a}	83.22 ± 3.08 ^{b}	74.23 ± 10.68 ^{a}	2.28 ± 0.12 ^{a}	17.15 ± 1.74 ^{a}
	100	117.26 ± 43.57	76.77 ± 46.76 ^{a}	91.49 ± 51.65	62.07 ± 15.52 ^{a}	82.62 ± 3.47 ^{b}	74.76 ± 16.01 ^{a}	2.18 ± 0.16 ^{a}	17.45 ± 3.90 ^{a}
	500	34.87 ± 30.30	4.21 ± 3.76 ^{b}	12.42 ± 10.81	8.11 ± 7.40 ^{b}	22.46 ± 19.68 ^{c}	23.86 ± 21.00 ^{b}	0.00 ± 0.00 ^{b}	0.00 ± 0.00 ^{b}
	0	99.00 ± 30.60 ^{a}	56.63 ± 39.58	67.89 ± 40.71 ^{a}	53.39 ± 23.42 ^{a}	80.38 ± 10.10 ^{a}	65.35 ± 20.92 ^{a}	1.91 ± 0.27 ^{a}	15.26 ± 4.14 ^{a}
	10	112.19 ± 51.93 ^{a}	71.01 ± 58.30	84.78 ± 57.80 ^{a}	57.42 ± 25.39 ^{a}	78.57 ± 15.20 ^{a}	71.28 ± 18.52 ^{a}	1.85 ± 0.49 ^{a}	11.55 ± 7.86 ^{a}
120	50	98.27 ± 50.82 ^{a}	65.72 ± 54.49	75.65 ± 56.21 ^{a}	60.65 ± 24.08 ^{a}	83.05 ± 10.32 ^{a}	71.79 ± 20.07 ^{a}	1.17 ± 1.19 ^{a}	10.27 ± 10.64 ^{a}
	100	95.50 ± 8.43 ^{a}	42.17 ± 32.53	59.43 ± 23.24 ^{a}	42.82 ± 30.28 ^{a}	65.24 ± 29.22 ^{a}	61.40 ± 18.92 ^{a}	1.36 ± 1.13 ^{a}	10.84 ± 12.15 ^{a}
	500	23.93 ± 33.84 ^{b}	2.15 ± 3.04	8.03 ± 11.35 ^{b}	4.48 ± 6.34 ^{b}	13.37 ± 18.90 ^{b}	16.77 ± 23.72 ^{b}	0.00 ± 0.00 ^{b}	0.00 ± 0.00 ^{b}

$CdCl_2$-cadmium chloride, VCL-curvilinear velocity, VSL-straight-line velocity, VAP-average-path velocity, LIN-linearity, STR-straightness, WOB-wobble, ALH-amplitude of lateral head displacement and BCF-beat-cross frequency, a-c = values in columns with different superscript letters were significantly different among the concentrations of $CdCl_2$ ($P<0.05$).

Table S15: Effect of CuSO4 and CdCl2 on sperm vitality percentages (mean ± SD) of vervet monkey (n = 3-4) after 60–180 min incubation.

Time (min)	$CuSO_4$ (μg/ml) n=4	Absorbance	$CdCl_2$ (μg/ml) n=3	Absorbance
	0	0.131 ± 0.033	0	0.098 ± 0.020
120	10	0.087 ± 0.042	10	0.086 ± 0.007
	50	-0.024 ± 0.022	50	0.050 ± 0.057
	100	-0.019 ± 0.023	100	0.043 ± 0.061

	250	0.007 ± 0.010	500	0.071 ± 0.013
	0	0.205 ± 0.034[a]	0	0.151 ± 0.021[a]
	10	0.150 ± 0.062[b]	10	0.132 ± 0.018[b]
180	50	0.006 ± 0.010[b]	50	0.081 ± 0.062[c]
	100	0.004 ± 0.009[b]	100	0.072 ± 0.062[c]
	250	0.008 ± 0.010[b]	500	0.086 ± 0.014[d]

a, b, c, d = values in columns with different superscript letters were significantly different among the concentrations of $CuSO_4$ and $CdCl_2$ ($P < 0.05$).
